# Assessing the relative importance of key quality of life dimensions for people with and without a disability: an empirical ranking comparison study

**DOI:** 10.1186/s12955-021-01901-x

**Published:** 2021-12-14

**Authors:** Matthew Crocker, Claire Hutchinson, Christine Mpundu-Kaambwa, Ruth Walker, Gang Chen, Julie Ratcliffe

**Affiliations:** 1 Health and Social Care Economics Group, Caring Futures Institute, Flinders University, Adelaide, GPO Box 2100, Adelaide, SA 5001 Australia; 2 Disability and Community Inclusion, College of Nursing and Health Sciences, Flinders University, Adelaide, GPO Box 2100, Adelaide, SA 5001 Australia; 3 Centre for Health Economics, Monash University, Melbourne, Melbourne, Caulfield East, VIC 3145 Australia

**Keywords:** Health economics, Economic evaluation, Disability, Preferences, Quality of life

## Abstract

**Background:**

In economic evaluation, the quality of life of people with a disability has traditionally been assessed using preference-based instruments designed to measure and value quality of life. To provide robust measurement of the effectiveness of programs designed to improve the quality of life of people living with a disability, preference-based measures need to be sufficiently sensitive to detect incremental changes in the quality of life dimensions that are most important to people who have a disability. This study sought to explore whether there was a difference in the ranked order of importance of quality of life dimensions between people with a disability and people without a disability.

**Methods:**

An online survey was developed and administered Australia wide. The first sample (n = 410) comprised adults (aged ≥ 18 years) with a disability (n = 208) and family carers of person/s with a disability who were asked to respond on behalf of the person with a disability (n = 202). The second sample included adults without disability (n = 443). Respondents were asked to rank the importance of 12 quality of life dimensions extracted from the content of established preference-based quality of life measures (EQ-5D, AQoL and ASCOT).

**Results:**

People with a disability placed relatively higher importance on broader quality of life dimensions (e.g. *Control, Independence, Self-care*) relative to health status focused dimensions (e.g. *Vision, Hearing, Physical mobility*). This distinction was less differentiable for those ‘without a disability’. The biggest differences in ranked importance of dimensions were in: *Vision* (‘with disability’ = 10th, ‘without disability’ = 4th), *Self-care* (‘with disability’ = 3rd, ‘without disability’ = 7th) and *Mental well-being* (‘with disability’ = 6th, ‘without disability’ = 2nd).

**Conclusions:**

The relative importance of quality of life dimensions for people with a disability differs to people without a disability. Quality of life is a key outcome for economic evaluation and for assessing the impact of disability care policy and practice in Australia and internationally. It is important that the effectiveness of interventions is measured and valued in ways which are fully reflective of the quality of life preferences of people with a disability.

## Background

The introduction of personalised budgets for people with assessed care needs due to their disability, is the most significant policy change introduced as a part of Australia’s National Disability Insurance Scheme (NDIS) [1]. This significant policy change follows similar moves to promote personalisation and consumer directed care in the disability care sectors of other countries, including the United Kingdom, the USA, Canada and the Netherlands [2]. Personalisation aims to empower consumers (people with a disability and family carers) to exercise choice and control over the care and support that they receive, and to make decisions about how their allocated funding is spent. Detailed consideration of the cost effectiveness of alternative services and supports through the application of an economic evaluation framework can provide a valuable resource for consumers and disability service providers to make informed choices about their care. Necessarily, the measurement of costs plays an important role in determining the value for money of services and supports. However, the measurement and valuation of quality of life also forms an important component of this process as it is the main outcome measure used in economic evaluation [3].

The challenge to accurately measure a person’s quality of life has been a longstanding issue for health economists [4]. Economic evaluation focuses on assessing the efficiency of resource allocation in terms of how different distributions of resources affect the quality of life of the population under consideration. To compare the effectiveness of different interventions in this context, generic preference-based measures of quality of life have become the preferred method of assessment [5], whereby individual responses to a measure can be converted into a summary quality of life score or utility score using the preference-based scoring algorithm relevant to that measure. Scoring algorithms are typically developed from the quality of life preferences of large general population based samples; the majority of whom are living without disability [3].

One of the most widely used generic preference-based measures internationally is the EuroQoL Five Dimension (EQ-5D) tool. It has been used extensively to measure health related quality of life in patient population samples and in large population-based studies [10, 11]. Another generic preference-based measure, which has been widely utilised in a similar context in Australia, is the Assessment of Quality of Life Four Dimension (AQoL-4D). Whilst both of these measures have a generic focus and application, the Adult Social Care Outcome Toolkit (ASCOT) was developed specifically for application in social care to capture the effect that social care-related needs (not just health-related needs) have on an individual’s quality of life [6]. The ASCOT was developed with a sample of UK social care services including people with long-term health conditions, physical disability or sensory impairment, mental illness, intellectual or developmental disability, or age-related needs [6]. These three preference based instruments (EQ-5D, AQoL-4D and ASCOT) provide an opportunity to compare the relative importance of health-related and social-care related dimensions of quality of life between people with and without a disability.

Recent developments in the measurement and valuation of quality of life have acknowledged the need to consider both health and broader dimensions of quality of life. This has led to the development of new generic preference-based measures including the ICEpop CAPability measures (ICECAP) [7] and the EuroQoL Health and Wellbeing (EQ-HWB) [8] instruments, designed to capture broader quality of life and wellbeing benefits beyond health status.

Refining preference-based measures to more accurately reflect the quality of life preferences important to different populations will continue to be an important area of development within health economics [3].

As the world’s population continues to age, more and more people will fall under the World Health Organisation’s definition of disability [9]: an umbrella term for impairments of body function or structure, activity limitations or participation restrictions [10]. In 2020, 4.4 million Australians (18% of the population) were living with a disability [10]. Eligibility for funding in Australia’s NDIS requires the person to have a permanent impairment (e.g. physical, intellectual, cognitive, neurological, visual, hearing or psychosocial) that results in significant disability [11]. If Governments are to be effective in providing care and support in schemes such as the NDIS that aim to maximise the quality of life of people with disabilities, it is important that their perspectives are considered and incorporated into outcome metrics for quality assessment and economic evaluation.

Whilst there is a developing literature which has documented differences in empirical rankings of a series of common quality of life domains between younger and older people [12–14], less is known about the relative importance that people living with a disability place on alternative quality of life dimensions. Although there is evidence to suggest that carers’ evaluation of the quality of life of the person whom they care for, diverges from that person’s self-reported quality of life [15–17], there is little research which has explored if there is a difference in the ranked importance of different quality of life dimensions between those with and without a disability [6]. This study aimed to investigate whether there was a difference in the empirical rankings of a series of key quality of life dimensions incorporated in three preference-based instruments (EQ-5D, AQoL-4D, ASCOT) between two populations: disability (people with a disability and family carers of people with a disability) and without disability (general population no disability).

## Methods

The main aim of this study was to investigate the relative importance rankings of 12 key quality of life dimensions mapped from three commonly applied preference-based measures (EQ-5D, AQoL-4D and the ASCOT) between population samples with and without a disability. Two, separate online surveys were developed. The first survey was developed for administration to people with a disability or family carers of people with a disability (where the person was unable to respond for themselves). The second survey was developed for administration to a general population sample of people without a disability. Both surveys were administered by Pureprofile—an online panel company. Members of Pureprofile (a panel comprising over 452,000 Australian adults living with and without disability in the community) were invited by the online panel company to complete either survey. The disability status of a person is unknown to Pureprofile and members were invited based on obtaining samples representative of Australian population age group, gender and Australian State of residence normative values. Eligible respondents completed the survey after providing informed consent. The first survey (‘general population’) was administered to a general population sample. Eligible respondents were Australian adults aged 18 years and older who were able to read and respond to a survey in the English language. The second survey (‘with disability’) was also administered to a general population sample of Australian adults aged 18 years, but respondents were screened out from completing the survey if they did not identify as having some form of disability or did not identify as the formal carer of a person with a disability who was unable to respond for themselves (e.g. due to severe physical and/or intellectual impairment). The carers were instructed to complete the survey from the person’s perspective, thinking about how the person who they care for would respond if they were able to do so. Survey respondents were paid between $2–10 AUD from Pureprofile, dependent on the length of time taken to complete the survey.

**Fig. 1:**
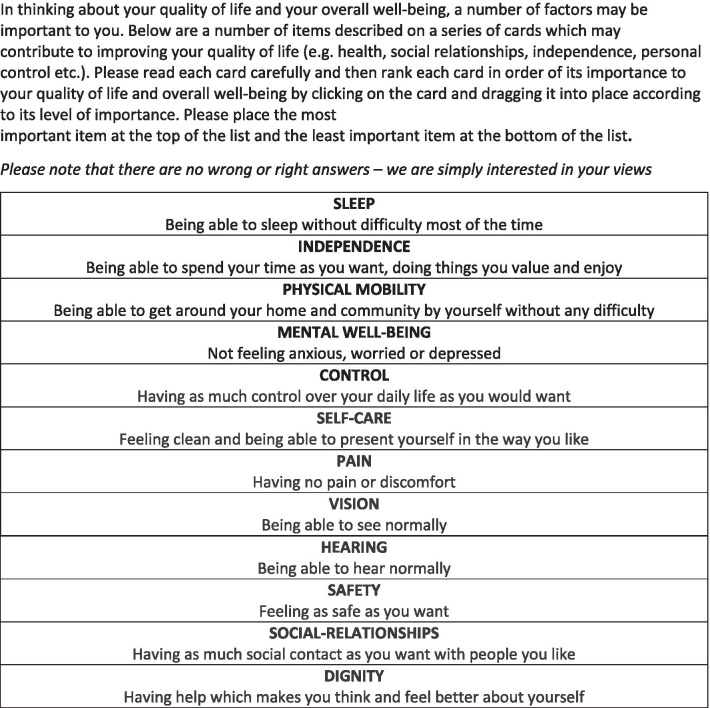
Quality of life dimensions ranking task

The two surveys were identical except for the introductory section that screened respondents’ eligibility to complete the ‘with disability’ survey. Each survey consisted of four main sections. In Section A respondents were asked to complete the ASCOT (four-level self-completion questionnaire) and AQoL-4D measures. To reduce the potential for ordering effects, the order of which survey measure appeared first was randomised within each survey group. Section B asked the survey respondents to rank a list of 12 quality of life dimensions in order of their importance to their quality of life. The 12 quality of life dimensions were extracted from the content of the EQ-5D, the AQoL-4D and the ASCOT instruments. Across the three preference-based measures there were 25 items. The research team consulted with stakeholders in the disability sector (including consumers and service providers) to identify 12 dimensions that were thought to reflect health-related and broader health-related quality of life dimensions important to people. The number of dimensions was limited to 12 as we wanted to include a balance of health-related and broader health-related dimensions of quality of life to detect differences between the population samples, but we were also conscious of not making the ranking task too burdensome. The results of the mapping exercise can be found in Appendix: Table 6. To reduce the potential of any ordering effects, the order in which the quality of life dimensions were presented in Section B was randomised for each survey respondent across both surveys. Respondents were asked to drag and drop a total of 12 quality of life dimension cards (Fig. 1) in order of their relative importance in determining their overall quality of life. The first subset of quality of life dimensions were more specifically focused on health status (e.g. physical mobility, pain, mental health, sleep, hearing, vision). The second subset were classified as dimensions which may affect an individual’s broader quality of life (e.g. independence, self-care, control, social relationship, safety, dignity). Section C comprised of a series of brief socio-demographic questions. The final section of the survey (Section D) asked respondents to indicate how difficult they found the survey to complete on a scale from 1 to 4, where 1 indicated ‘not difficult’ and 4 indicated ‘very difficult’.

### Exclusion criteria

To compare the relative importance rankings of the quality of life dimensions between the two surveyed groups, all responses from the ‘general population’ survey were screened to generate the general population without disability group. Respondents were excluded if they indicated a ‘yes’ response to one or more of the following questions.

- Do you have a long-term disability, illness or medical condition?
- Do you need any help looking after yourself?
- When doing household tasks (e.g. cooking, cleaning) do you need any help?
- Do you need any help to get around your home and community?
- Do you currently receive any care support or services which enable you to remain living at home?

### Socio-economic status

The socio-economic status of survey respondents (‘with disability’ and ‘without disability’ sample populations) was calculated according to the Australian Bureau of Statistics’ Socio-Economic Indexes for Areas (SEIFA) [18]. The SEIFA is a group of indexes which ranks the geographic area in which the individual resides according to details obtained from the Australian Census of Population and Housing [18]. According to their residential postcode, respondents from both surveyed groups were assigned a SEIFA Index of Relative Socio-economic Advantage and Disadvantage score [19]. Respondents were then placed into the following groups of advantage according to their score: Low (1–4), Medium (5–7) and High (8–10) [18, 19].

### Analysis

All data analysis was completed in R version 4.0.3 [20]. Descriptive summary statistics and between group differences were calculated. Normality was tested for all data using the Shapiro-Francia normality test and statistically significant differences between categorical groups were explored using Pearson’s Chi-squared test. For continuous variables, the Wilcoxon rank-sum test with continuity correction was used to assess for differences between groups [21]. To assess the similarities between rankings of the 12 quality of life dimensions, intraclass correlation coefficient (ICC) estimates and their 95% confidence intervals were calculated based on a two-way random effects model [22]. Average ICCs (estimated correlations between average rankings made on the same quality of life dimensions by different rankers) were estimated as we were interested in the level of agreement amongst raters as to the ranked position of each quality of life dimension for the ‘with and without disability’ groups [21]. An ICC under 0.5 was interpreted as being indicative of poor interrater reliability, values between 0.5 and 0.75 were classified as moderate reliability, 0.75 to 0.9 good reliability, and an ICC greater than 0.9 indicated excellent reliability [22]. A high average ICC was interpreted as indicating a high level of agreement amongst raters as to the ranked position of each quality of life dimension.

The quality of life dimensions were ranked using a similar approach to that adopted by Ratcliffe et al. [14] which compared the quality of life preferences of younger (aged 18–64) and older people (aged 65 years and above). For example, a quality of life dimension that was ranked as the most important factor by a survey respondent, received a score of 12 points. The second most important dimension was assigned 11 points, and this pattern occurred until the 12th ranked quality of life dimension received 1 point. The total number of points allocated to each quality of life dimension was summed for each group (with and without disability) and then presented as a percentage of the total number of available points. This allowed for direct comparison between the preference orderings of people with disability and without disability.

## Results

For the ‘general population’ survey, 2124 individuals were approached, of whom 1532 (72%) consented to participate and 1000 fully completed the survey (47% of those initially approached). As per the exclusion criteria, 557 respondents were removed from the ‘general population’ group which left 443 survey respondents who were classified as the ‘without disability’ group population.

For the ‘with disability’ survey, 3538 persons provided informed consent, of which 2988 (84%) were screened out as they did not identify as having a disability or as providing care for someone with a disability. A further 140 (4%) started the survey but did not complete it. A total of 410 people (11% of those who provided consent) fully completed the survey.

Data from the ‘with disability’ (n = 410) and ‘without disability’ (n = 443) surveys were combined. A dummy variable (1 = with disability) was assigned to the combined group to identify respondents who have a disability (or who were responding on behalf of someone with a disability).

The ‘with disability’ group consisted of proxy (n = 202) and self-reported (n = 208) responses. The characteristics of those with a disability who answered the survey themselves (self-reported) and the characteristics of the person with a disability who had their carer report on their behalf (proxy-reported) were similar. With respect to disability characteristics, there was no statistically significant difference between the self-reported and proxy-reported sub-groups: Onset of disability (X^2^ = 2.92, *p* = 0.09) and Type of disability (X^2^ = 5.57, *p* = 0.06) (Table 1).

**Table 1:** Characteristics of the ‘with disability’ group

Characteristics	Self-reported (n = 208)	Proxy-reported (n = 202)	With disability (n = 410)	Test of difference (self vs proxy-reported)
Onset of disability: n (%)				
Birth	29 (13.94)	41 (20.30)	70 (17.07)	X^2^ = 2.92, *p* = 0.09
Acquired	179 (86.06)	161 (79.70)	340 (82.93)	
Type of disability: n (%)				
Intellectual	37 (17.79)	47 (23.27)	84 (20.49)	
Physical	136 (65.38)	109 (53.96)	245 (59.76)	X^2^ = 5.57, *p* = 0.06
Both intellectual and physical	35 (16.83)	46 (22.77)	81 (19.76)	

X^2^ is a Chi-squared test

To justify the pooling of the responses within the ‘with disability’ group (i.e. treating self and proxy-reported responses as one group rather than two separate ones), it was important that irrespective of who responded (proxy or self-reported) there was a high average ICC within the pooled group. A high average ICC indicated that the quality of life preferences reported by the proxies were like the self-reported preferences.

Table 2 presents the ICC statistics. The average ICCs (averages of rankings) for the same quality of life dimension among several rankers in both groups were greater than 0.9, denoting excellent reliability (‘with disability’ = 0.989 and ‘without disability’ = 0.987). The average ICC was also calculated for the ‘with disability’ sub-groups: self-reported = 0.976 and proxy-reported = 0.983. The average ICC for the entire ‘with disability’ sample was larger than the average ICCs of the ‘with disability’ sub-group analyses. This indicated a very high level of interrater reliability within the ‘with disability’ sample allowing the ‘with disability’ and ‘proxy’ samples to be pooled and analysed as a single sample. The high average ICCs indicate a high level of agreement as to the ranked position of the quality of life dimensions within the ‘with disability’ and ‘without disability’ groups.

**Table 2:** Absolute agreement Intraclass Correlation (ICC) for rankings of 12 quality of life dimensions

Type	Group	ICC	95% Confidence interval		F test with true value = 0			
			Lower bound	Upper bound	Value	df1	df2	*p*-value
Average^a^	With disability (n = 410)	0.989	0.979	0.996	86.7	11	4499	< 0.000
	Without disability (n = 443)	0.987	0.987	0.995	68.9	11	4862	< 0.000
	Sub-group analysis							
	Self-reported (n = 208)	0.976	0.951	0.992	37.5	11	2277	< 0.000
	Proxy-reported (n = 202)	0.983	0.966	0.994	53.4	11	2211	< 0.000

ICC calculation performed in R version 4.0.3 using the package irr().Average rating, absolute agreement, twoway random effects

^a^Agreement between the average ranking on the same QUALITY OF LIFE dimension by different survey respondents

The characteristics for each group are presented in Table 3. The mean age for the ‘with disability’ and ‘without disability’ groups was very similar: 53.6 (18.5) and 52.7 (17.9) years respectively. Similarly, there was no statistically significant difference in the distribution of ages between the groups (X^2^ = 8.35, *p* = 0.14) and there was approximately an equal proportion of women in both groups (X^2^ = 0.67, *p* = 0.41). Differences in self-reported levels of general health were observed between the two groups with people with a disability reporting lower levels of health relative to people without disability, and these differences were found to be statistically significant (X^2^ = 366.63, *p* < 0.001). In the ‘with disability’ group, 58% of respondents (n = 236) indicated that their health was ‘Fair’ or ‘Poor’. In comparison, 71% of respondents (n = 312) in the ‘without disability’ group identified that their health was ‘Excellent’ or ‘Very good’. There was no statistically significant difference between the groups’ levels of socio-economic advantage according to SEIFA classifications (X^2^ = 2.50, *p* = 0.29).

**Table 3:** Respondent characteristics according to respondent group

Characteristics	With disability (n = 410)	Without disability (n = 443)	Total sample (n = 853)	Test of difference (with vs without disability)
Age in years				
Mean (SD)	53.6 (18.5)	52.7 (17.9)	53.1 (18.2)	Z* = 90,723, *p* = 0.98
Median (IQR)	56 (38, 66)	55 (36, 68.5)	56 (37, 68)	
Age group: n (%)				
18–29	50 (12.20)	62 (14.00)	112 (13.13)	
30–39	56 (13.66)	74 (16.70)	130 (15.24)	
40–49	61 (14.88)	56 (12.64)	117 (13.72)	X^2^ = 8.35, *p* = 0.14
50–59	65 (15.85)	46 (10.38)	111 (13.01)	
60–69	91 (22.20)	112 (25.28)	203 (23.8)	
70+	87 (21.22)	93 (20.99)	180 (21.1)	
Gender: n (%)				
Female	205 (50)	208 (46.95)	413 (48.42)	X^2^ = 0.67, *p* = 0.41
Health status^: n (%)				
Excellent	5 (1.22)	103 (23.25)	108 (12.66)	
Very good	53 (12.93)	209 (47.18)	262 (30.72)	
Good	116 (28.29)	112 (25.28)	228 (26.73)	X^2^ = 366.63, *p* < 0.001
Fair	168 (40.98)	17 (3.84)	185 (21.69)	
Poor	68 (16.59)	2 (0.45)	70 (8.21)	
SEIFA deciles^+^: n (%)				
Low (1–4)	144 (35.12)	134 (30.25)	278 (32.71)	
Medium (5–7)	110 (26.83)	124 (27.99)	234 (27.43)	X^2^ = 2.50, *p* = 0.29
High (8–10)	154 (37.56)	184 (41.53)	338 (39.62)	

^*^Wilcoxon rank-sum test with continuity correction; X^2^ is a Chi-squared test

^^^Self-reported health status

^+^Socio-Economic Indexes for Areas ranks areas within Australia relative to socio-economic advantage and disadvantage. Postcode data was missing for two respondents in ‘with disability’ and one respondent in ‘without disability’

Table 4 presents the ranking of quality of life preferences by the percentage of points allocated to each quality of life dimension. If a dimension was ranked 1st it received 12 points, 2nd 11 points, and so on until the 12th ranked dimension received 1 point. The order of preference rankings between the two groups differed, which suggests that the presence of a disability affects what factors are important to someone’s quality of life. For the ‘with disability’ group the most important quality of life dimension was *Control* (received 10.91% of available points), whereas *Control* was ranked 4th (9.74%) for the ‘without disability’ group. In terms of ranked position based on proportion of total points allocated, the biggest differences between the groups were for the quality of life dimensions *Vision* (6 ranking places) and *Physical mobility* (4 ranking places). In both instances, the ‘with disability’ group valued these quality of life dimensions less than the ‘without disability’ group. There was agreement in the ranked order for *Pain* and *Sleep*. The preferences of the ‘with disability’ group were determined by self-reported (person with a disability) and proxy-reported (carer of person with a disability) groups. The two furthest columns of Table 4 demonstrate that there was a large degree of agreement between self-reported and proxy-reported preferences in the ‘with disability’ group. The largest differences were for *Pain* (ranked 2 places higher by self-reported) and *Dignity* (ranked 2 places lower by self-reported).

**Table 4:** Quality of life dimensions ranked by proportion (%) of available points allocated

Rank	Without disability (%) (n = 443)	With disability (%) (n = 410)	With disability (n = 410)	
			Self-reported (%) (n = 208)	Proxy-reported (%) (n = 202)
1	Independence (11.19)	Control (10.91)	Control (10.83)	Control (10.99)
2	Physical mobility (10.59)	Independence (10.67)	Independence (10.64)	Independence (10.69)
3	Mental well-being (10.11)	Self-care (9.83)	Mental well-being (9.78)	Self-care (10.41)
4	Control (9.74)	Mental well-being (9.65)	Self-care (9.28)	Safety (9.91)
5	Vision (8.78)	Safety (9.51)	Safety (9.12)	Mental well-being (9.53)
6	Self-care (8.03)	Physical mobility (8.58)	Pain (8.78)	Physical mobility (8.5)
7	Pain (7.38)	Pain (8.21)	Physical mobility (8.65)	Dignity (8.02)
8	Safety (7.4)	Social relationships (7.63)	Sleep (7.56)	Social relationships (7.96)
9	Social relationships (7.14)	Dignity (7.60)	Social relationships (7.31)	Pain (7.62)
10	Sleep (6.82)	Sleep (7.13)	Dignity (7.19)	Sleep (6.68)
11	Hearing (6.61)	Vision (5.69)	Vision (6.05)	Vision (5.33)
12	Dignity (6.21)	Hearing (4.58)	Hearing (4.81)	Hearing (4.34)

Sample variance of the proportion of points allocated to each quality of life dimension was larger in the ‘with disability’ group (S^2^ = 3.74), than the ‘without disability’ group (S^2^ = 2.91). A larger sample variance indicated that there was more agreement in the rank order of quality of life dimensions within the ‘with disability’, group as specific dimensions consistently received more (or less) points. The consistency of how the points were allocated, resulted in a larger sample variance in the ‘with disability’ group.

**Fig. 2:**
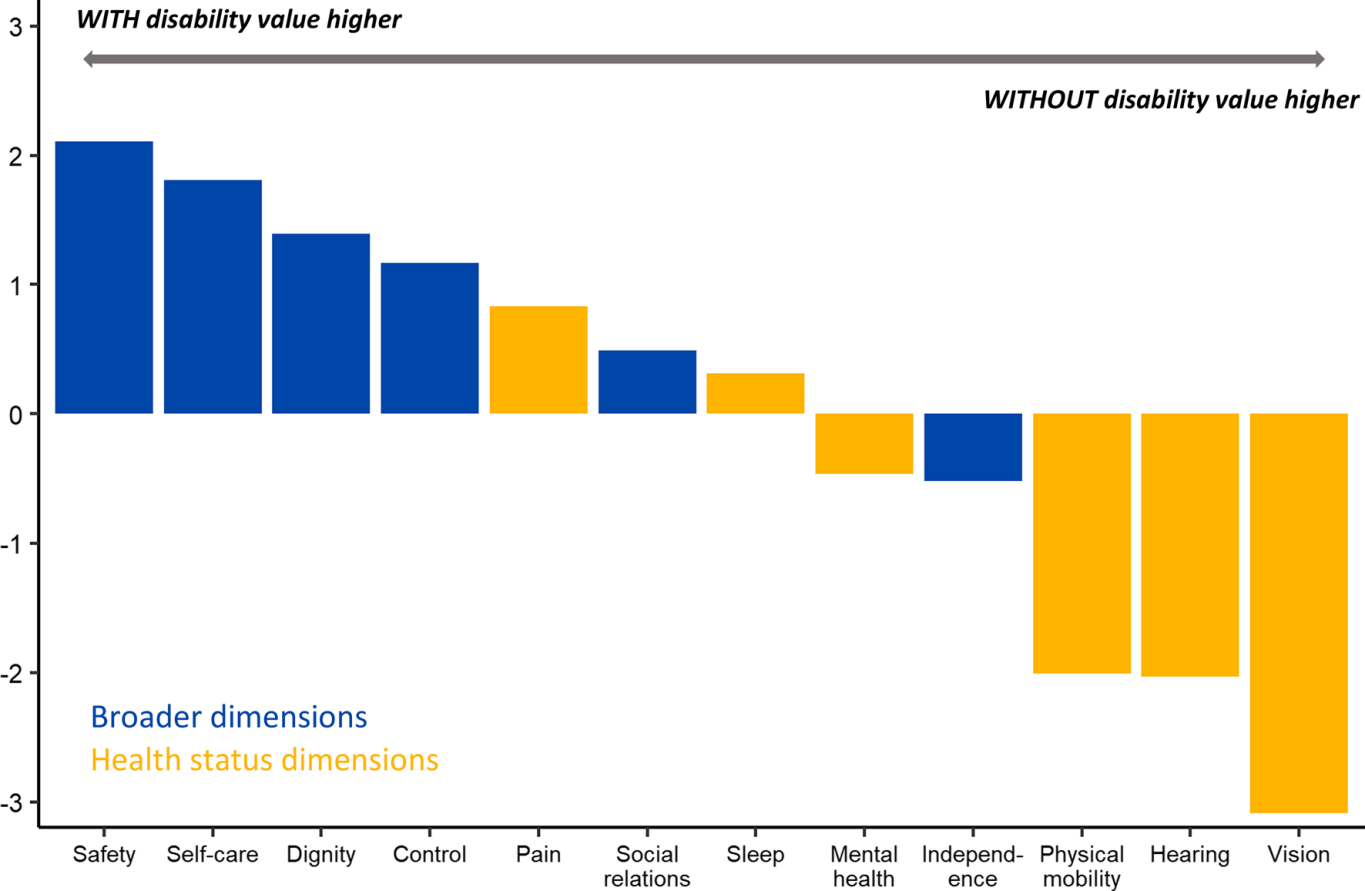
Percentage point difference in the relative importance of quality of life dimensions by disability status. *Note*: Percentage point difference shown (‘with disability’—‘without disability’)

Figure 2 provides a visual representation of the differences between the groups in the proportion of points allocated to each quality of life dimension. Comparison of the proportion of points allocated indicates the relative weight assigned to each quality of life dimension and provides insight into the magnitude of differences between the two groups. The dimension *Vision* had the largest ranking difference between groups (ranked more highly by the ‘without disability’ group), and also returned the greatest difference in preference weighting (3.09% points). The second largest percentage point difference was for the dimension *Safety*. Those from the ‘with disability’ group allocated 2.11 more percentage points to this dimension than those ‘without disability’; clearly demonstrating that safety is of more importance to the quality of life of someone with a disability compared to someone without a disability. The only other dimensions where there was a ≥ 2% point difference in point allocation were in *Hearing* and *Physical mobility*. For both dimensions, the ‘without disability’ group identified these dimensions as more important to their quality of life than the ‘with disability’ group. Despite only a one rank position difference between groups for *Hearing* (11th vs 12th), there was a 2.03 percentage point difference in the proportion of points allocated. For the ‘with disability’ group, there was a consensus that *Hearing* is not of high importance to their quality of life, whereas for those without a disability, although ranked lowly, there was less agreement within the group as to its importance to their quality of life. Figure 2 demonstrates that there is a difference in how people with and without a disability preference different quality of life dimensions. People with a disability more highly value broader dimensions of quality of life than health status dimensions.

Table 5 presents the frequency of respondents who ranked each of the 12 quality of life dimensions 1st; 1st or 2nd; 1st, 2nd or 3rd; and 1st, 2nd, 3rd or 4th. Across both ‘with disability’ and ‘without disability’, the number one ranked quality of life dimension did not change position across the ranking groups (e.g. Rank 1; Rank 1 or 2; Rank 1, 2 or 3; Rank 1, 2, 3 or 4). For ‘with disability’, *Control* occupied the first ranked position across all groups, whereas for ‘without disability’ *Independence* remained the most popular dimension across all ranking groups.

**Table 5:** Count frequency: quality of life dimension rankings summary (up to first four)

Dimension	Rank 1				Rank 1 or 2				Rank 1, 2 or 3				Rank 1, 2, 3 or 4			
	With disability		Without disability		With disability		Without disability		With disability		Without disability		With disability		Without disability	
	n	%	n	%	n	%	n	%	n	%	n	%	n	%	n	%
Broader quality of life dimensions																
Control	100	24.39	53	11.96	155	18.90	103	11.63	204	16.59	154	11.59	239	14.57	198	11.17
Independence	68	16.59	132	29.80	138	16.83	197	22.23	185	15.04	235	17.68	236	14.39	273	15.41
Self-care	49	11.95	20	4.51	104	12.68	38	4.29	160	13.01	78	5.87	187	11.40	122	6.88
Safety	43	10.49	23	5.19	90	10.98	47	5.30	145	11.79	75	5.64	184	11.22	104	5.87
Social relationships	15	3.66	15	3.39	42	5.12	48	5.42	67	5.45	78	5.87	110	6.71	109	6.15
Dignity	14	3.41	11	2.48	35	4.27	26	2.93	63	5.12	47	3.54	98	5.98	81	4.57
Total	289	70.49	254	57.34	564	68.78	459	51.81	824	66.99	667	50.19	1054	64.27	887	50.06
Health status dimensions																
Pain	43	10.49	28	6.32	69	8.41	60	6.77	89	7.24	90	6.77	119	7.26	121	6.83
Mental well-being	40	9.76	53	11.96	83	10.12	105	11.85	141	11.46	158	11.89	192	11.71	211	11.91
Physical mobility	17	4.15	52	11.74	45	5.49	123	13.88	75	6.10	189	14.22	126	7.68	243	13.71
Sleep	8	1.95	15	3.39	27	3.29	31	3.50	53	4.31	54	4.06	79	4.82	73	4.12
Vision	8	1.95	31	7.00	19	2.32	76	8.58	29	2.36	114	8.58	43	2.62	159	8.97
Hearing	5	1.22	10	2.26	13	1.59	32	3.61	19	1.54	57	4.29	27	1.65	78	4.40
Total	121	29.51	189	42.66	256	31.22	427	48.19	406	33.01	662	49.81	586	35.73	885	49.94
Test of difference^a^	Z^b^ = -4.842, *p* = 0.000				Z^b^ = -7.351, *p* = 0.000				Z^b^ = -8.512, *p* = 0.000				Z^b^ = -8.171, *p* = 0.000			
(with vs without disability)																

^a^Two-sample Wilcoxon rank-sum (Mann–Whitney) test

^b^Z = Z statistic

How both groups ranked the ‘Broader quality of life dimensions’ and ‘Health status dimensions’ was different between the ‘with disability’ and ‘without disability’ groups. Across all four ranking columns, greater than 60% of respondents in the ‘with disability’ group included a ‘Broader quality of health dimension’ as either their 1st, 2nd, 3rd or 4th preference. The ‘Rank 1’ column shows the biggest split between ‘Broader quality of life dimensions’ (70.49%) and ‘Health status dimensions’ (29.51%) for the ‘with disability’ group, and this difference continued largely into the final ranking group (64.27% vs 35.73%). A persistent preference for one type of quality of life dimension was less apparent across the ranking columns for ‘without disability’. Rank 1 column shows a clear preference for ‘Broader quality of life dimensions’ (57.34% vs 42.66%), but this all but disappeared by the last ranking group (50.06% vs 49.94%). The absence of an obvious difference between broader and health status quality of life dimensions in the ‘without disability’ group suggests that those without a disability do not have a clear preference for a type of quality of life dimension. In contrast, the ‘with disability’ group demonstrated a clear preference for broader quality of life dimensions.

## Discussion

The main findings from this study indicate that people with a disability place relatively higher importance on quality of life dimensions which can be categorised as broader quality of life dimensions, relative to health status focused dimensions. Although the ‘without disability’ group ranked *Independence* and *Control* as their 1st and 2nd most important quality of life dimensions (same as the ‘with disability’ group), when the ranking order of all 12 dimensions was compared between groups, it was clear that the ‘with disability’ group placed more importance overall on broader dimensions of quality of life. Application of the ranking points system which captured relative importance rankings across all 12 quality of life domains (Fig. 2), revealed that people with a disability placed relatively more importance on quality of life dimensions *Safety*, *Self-care* and *Dignity*, than people without a disability. These findings concur with several previous studies that have identified potential differences in the conceptualisation of quality of life for people with a disability relative to people without a disability [20–23]. A plausible explanation for this difference is adaptation, that is people may learn to live with the limitations of their current health state and adjust their expectations based on what aspects of their life they can realistically influence [24]. Ratcliffe et al. [14] found that older people tended to be more accepting of the limitations of their current health state and therefore placed more importance on broader quality of life domains than did younger people (who were more focused on health domains). A similar phenomenon may explain differences in quality of life preferences for people with and without a disability. However, it should be noted that our findings contrast with the conclusion of Netten et al. [6] who found that there was no statistically significant difference in how ‘service users’ and the ‘general population’ ranked the importance of the ASCOT’s dimensions. In developing the ASCOT, Netten et al. [6] employed discrete choice experiments and best–worst scaling techniques to explore if there were differences in preferences between ‘service users’ and the ‘general population’. A possible explanation for these differences is that Netten et al. [6] focussed specifically on social care related dimensions of quality of life, whereas this study included both health and broader dimensions of quality of life.

Identifying what aspects of quality of life are most important to people with a disability is an important step in the development of preference-based instruments which are designed to capture their preferences. Townsend-White et al. [25] conducted a systematic review of 24 quality of life measures designed to measure changes in the quality of life of people with intellectual disabilities. Six quality of life measures were identified as being psychometrically sound. However, the authors concluded that there is not a universally accepted ‘gold-standard’ for measuring the quality of life of adults with an intellectual disability [25]. A recent systematic review by Bray et al. [26] explored which instruments are commonly used to assess the quality of life of individuals with congenital mobility disabilities. The authors also concluded that current quality of life instruments are insufficient to capture the quality of life dimensions of most importance to people with a disability [26]. It is important to note, however, that both systematic reviews did not specifically review preference-based quality of life measures and neither included the ASCOT measure; which was designed for assessing the social care-related quality of life of people with support needs related to long-term health conditions or disability. Another difference between these reviews and our research was that they each focussed on specific disability populations, unlike our research that did not differentiate by type of disability. The literature referred to here is illustrative of the ongoing work required to better measure the quality of life of people with disability and we acknowledge that development in this space is ongoing. For example, the recently developed ASCOT Easy Read (ASCOT-ER) is a good illustration of how existing quality of life measures are being refined to be more accessible for people with a disability [27]. Our research contributes to this agenda by offering preliminary findings that suggest that there is a difference in the relative ranking importance of quality of life dimensions between people with a disability and those without disability.

In this study we mapped quality of life dimensions from three commonly applied preference-based measures and investigated their relative importance to people with and without disability. Of the three measures, the ASCOT is the most relevant measure for people with a disability as it was developed with people who used social care services [6]. The empirical ranking exercise has drawn out the differences in relative importance rankings for alternative quality of life dimensions between those with and without disability. It may initially be surprising that the relative importance rankings of quality of life dimensions for people with a disability and their carers were broadly equivalent given the extensive body of literature that indicates that proxy reported quality of life can often differ markedly from self-reported quality of life [26–29]. However, this study was different in that it focused on the relative weights attached to different quality of life dimensions by self and proxy sub-groups, not on the actual quality of life of a person with a disability. As such it is perhaps less surprising that strong agreement was found in this context.

As with any study of this nature, there were several limitations which are important to highlight. The quality of life dimensions included in this study were not based upon an extensive review of potential quality of life dimensions. Of the three tools used to identify the 12 quality of life dimensions, two measures (EQ-5D and AQoL-4D) are generic and one (ASCOT) is more specifically focused on social care-related quality of life. The iterative consultative process used to determine the final 12 quality of life dimensions included, and their unique descriptions, may have influenced the relative importance rankings. For example, the definition for *Dignity* was based heavily on question eight of the ASCOT and it assumed that the respondent required “help to do things”. There is evidence to suggest that people find it difficult to perceive of a health status that they do not experience [3, 30] and therefore their ability to accurately rank a dimension that does not relate to their reality may be compromised. In addition, we cannot rule out that respondents did not bring their own interpretations of quality of life dimensions (independent of any definition provided) to the task. It is possible that relative importance rankings may have been different had different quality of life definitions been used.

Across both population groups, people from a higher socio-economic status were slightly more represented than those from lower socio-economic statuses. According to the 2016 Australian census, most Australians were classified as enjoying a medium level of advantage according to the SEIFA’s Index of Relative Socio-economic Advantage and Disadvantage score [31]. Respondents were recruited via an online panel, survey company (Pureprofile). Whilst Pureprofile’s panel membership base is large and diverse, individuals registered to Pureprofile are not entirely representative of Australia’s population and are likely to be more computer literate than the general Australian population. In addition, survey respondents were required to self-report whether they had a disability. Whilst there was no significant monetary incentive within this study to self-report having a disability, we cannot be sure that the ‘with disability’ group consisted entirely of people with a disability and family carers of a person with a disability. Further research should be directed at confirming the preliminary findings reported here in larger and more diverse samples of people with a confirmed disability (as opposed to relying on self-report) and across a larger set of quality of life dimensions.

Within Australia, the NDIS is expected to support over half a million people with a disability by the end of 2022–2023 [32]. To improve the ongoing evaluation of the NDIS (and other international disability insurance programs) it will be important to continue to improve and refine the tools used to measure and value quality of life to assess the effectiveness of interventions in policy and practice for people with a disability. This paper makes a valuable contribution by providing preliminary evidence to suggest that the quality of life dimensions important to a person with a disability are potentially different to a person without a disability. Those responsible for evaluating the NDIS or other disability insurance programs will need to be cognisant of these differences.

## Conclusions

In conclusion, this empirical study indicates that the relative importance rankings of key quality of life domains for people with a disability differ to those without a disability. In general, people with a disability place more importance on broader quality of life dimensions including *Safety, Self-care, Control* and *Independence*, than physical health attributes including *Vision, Hearing* and *Physical mobility*. Quality of life is a key outcome for economic evaluation and for assessing the impact of disability care policy and practice in Australia and internationally. It is important that the effectiveness of interventions to provide care and support for people with a disability is measured and valued in ways which are fully reflective of the key indicators of quality of life from the perspective of people with a disability.

## Data Availability

The datasets generated and analysed during this study are not publicly available due to Flinders University ethics requirements, but are available from the corresponding author on reasonable request.

## Appendix

See Table 6.

**Table 6:** Extraction of quality of life dimensions from the EQ-5D, AQoL-4D and ASCOT. Adapted from Ratcliffe et al. [14]

	EQ-5D	AQoL-4D	ASCOT
Health status dimensions			
Physical mobility	✓	✓	
Mental well-being	✓	✓	
Pain	✓	✓	
Sleep		✓	
Vision		✓	
Hearing		✓	
Well-being dimensions			
Control			✓
Self-care	✓	✓	✓
Independence	✓	✓	
Safety			✓
Social relationships		✓	✓
Dignity			✓

## Abbreviations

AQoL-4D: Australian Quality of Life 4 Dimension
ASCOT: Adult Social Care Outcomes Toolkit
ICC: Intraclass Correlation Coefficient
NDIS: National Disability Insurance Scheme
SEIFA: Socio-Economic Indexes for Areas
